# Learning and Instruction: How to Use Technology to Enhance Students’ Learning Efficacy

**DOI:** 10.3390/jintelligence12070064

**Published:** 2024-06-28

**Authors:** Gyöngyvér Molnár

**Affiliations:** MTA–SZTE Digital Learning Technologies Research Group, Institute of Education, University of Szeged, Petőfi S. sgt. 32-34, 6722 Szeged, Hungary; gymolnar@edpsy.u-szeged.hu

Due to the rapid development of technology (see, e.g., the potential of ChatGPT), it is predicted that 65% of children entering school today will be working in jobs that do not yet exist (British Council 2018). This prediction poses a serious challenge for education systems, educational researchers, schools, teachers, and students. The question thus arises: what can an effective school system do to optimally prepare students for the unknown in a rapidly changing world, to provide them with skills that will enable them to thrive in the workplace of the future and engage in lifelong learning? This question comes even as everyone’s lives have been affected by significant changes in the speed of global development and learning and instruction, such as the changes encountered in the last few years during the COVID-19 pandemic (Molnár and Hermann 2023). Learning and learning effectiveness, thinking and reasoning, the application and creation of new knowledge, and innovation have become key words for an effective school system.

As a result of these rapid technological advances, we should also ask questions about how we can best use technology to boost students’ learning efficacy and how we can effectively make up for the learning loss that may have occurred during distanced education in those uncertain times. Technology, big data, learning analytics, machine learning, and artificial intelligence, based on the latest research results from the discipline of learning sciences, offer new opportunities to enhance our understanding of the concept of learning and overcome the “one-size-fits-all approach” by personalising education to make learning more effective.

For the present Special Issue on learning and instruction, we sought papers which ideally combine these issues and focus on topics such as the future of learning and instruction research; the role of reasoning skills in learning and reshaping school learning in the 21st century; the future of personalised learning: artificial intelligence and adaptive learning; and the potential of using process data to make learning processes visible.

The different contributions published in this Special Issue can be grouped into the two major research areas of learning and instruction: (1) those seeking a more thorough understanding of learning processes, specifically (a) by using technology to develop innovative and/or new assessment instruments, (b) using process and log data collected through technology-based assessments to gain a better understanding of students’ thinking and learning processes, and (c) assessing the long-term impact of different skills and abilities on later school achievements within the confines of longitudinal data collections, as well as (2) those using technology to make learning more effective.

I will now briefly introduce the different contributions, starting with papers in the first research area: those striving for a more thorough understanding of learning processes. The papers by Simon et al. (2022) and Varga et al. (2022) demonstrate new computer-based assessment instruments for authentic assessments of visual communication skills, in the case of the first study, and morphological awareness, in the case of the second. Both attempt to map the development of each of these areas among primary students and discover their relationship to a particular type of intelligence (visual–spatial and linguistic, respectively). Wu and Molnár (2022) monitor students’ thinking processes by investigating the roles of inductive reasoning and combinatorial reasoning in problem-solving, using process data to identify students with different ways of thinking. Józsa et al. (2022), Janurik and Józsa (2022), and Ehlert et al. (2022) explore the long-term impact of preschool predictors, intelligence, the mothers’ education, early musical abilities, working memory, and attention, on future success in school. Their findings show that developing preschool skills, IQ, and musical skills is essential for long-term learning success and that intellectual abilities determine both children’s domain-general resources (such as working memory and attention) and domain-specific ones, resulting in different developmental trajectories for mathematical skills. Csíkos (2022) raises the question of whether it is possible for the very same cognitive processes to be both controlled and controlling. He stresses the role of metacognitive scaffolding as a powerful educational approach to school learning and instruction.

Five contributions examine the second research area: using technology to personalise learning and make it more effective. Suh and Ahn (2022) analyse children’s experiences with and attitudes toward the metaverse for learning-centred education to determine how closely linked this virtual environment is to the lives of elementary students. They conclude that almost all of the students in their study have had experience with the metaverse and consider it closely tied to their everyday life. Gu et al. (2022a) investigate the relationship between virtual and physical environments. They conclude that the virtual environment provides interactions and novel real-world experiences which positively influence students’ interest in learning and intention to learn compared to traditional teaching methods. Gu et al. (2022b) discuss the teaching effects of augmented reality (AR) technology in German instruction with the conclusion that learning German with AR picture books, satisfaction is the key construct that determines students’ learning states. Pásztor et al. (2022) introduce a game-based developmental programme for teaching inductive reasoning through mathematics and examine its efficacy among fifth-grade students. The training programme proves to be effective in general. Ökördi and Molnár (2022) and Szili et al. (2022) present empirical evidence of the closing of the learning gap that developed during the COVID-19 pandemic using well-designed online intervention programmes in mathematics and reading for 9-to-11-year-olds outside the classroom—even without the presence of the teacher.

In summary, all of these papers contribute significantly to the field of learning and instruction. They confirm that technology offers great opportunities to learn about learning and to make learning more challenging and effective though smart education—especially through personalised learning (Zhuang et al. 2023).

Finally, I would like to express my thanks to the editor and the managing editor for proposing this Special Issue and inviting me to act as the guest editor, as well as to the authors and reviewers of the articles published in this issue for their excellent contributions. I hope that the *Journal of Intelligence* readers will find it as valuable and interesting as I do.
